# Development and Validation of an Interactive Game-Based Digital Cognitive Assessment Tool

**DOI:** 10.1093/geroni/igaf122.4098

**Published:** 2025-12-31

**Authors:** Ruike Sun, Huixian Li, Yanyan Wang

**Affiliations:** West ChinaHospital, Sichuan University, Chengdu, Sichuan, China (People’s Republic); West ChinaHospital, Sichuan University, Chengdu, Sichuan, China (People’s Republic); West ChinaHospital, Sichuan University, Chengdu, Sichuan, China (People’s Republic)

## Abstract

Early screening and intervention are essential for mitigating cognitive decline, particularly for the prevention of Alzheimer’s disease (AD) through timely detection of mild cognitive impairment (MCI). Despite advances in digital assessments, barriers still limit their adoption among older Chinese adults. This study developed a digital cognitive assessment tool integrating gaming scenarios and Chinese cultural elements for screening MCI in China (IGD-CAT) and evaluated its concurrent and discriminative validity. A theoretical framework was established through literature review, expert panel, and Delphi survey, and IGD-CAT was created via interdisciplinary collaboration. A total of 218 participants from communities and hospitals completed traditional assessments followed by IGD-CAT and were classified as normal cognition (NC) or MCI. Concurrent validity was examined through correlations with Montreal Cognitive Assessment (MoCA) scores, and discriminatory validity was assessed using a random forest model. IGD-CAT comprises 14 tasks across seven domains: time orientation, attention, memory, language, calculation, visuospatial ability, and executive function. Among 196 participants (mean age 63.6 ± 5.81 years), 93 were classified as MCI and 103 as NC. Task scores correlated positively with MoCA (r = 0.140–0.387), while completion times correlated negatively (r=-0.476 to -0.168). The total IGD-CAT score correlated with MoCA (r = 0.529, P<.001), whereas total completion time correlated negatively (r=-0.549, P<.001). In the test set, the random forest model achieved 89.65% sensitivity, 96.55% specificity, and an AUC of 0.96. IGD-CAT covers multiple cognitive domains, is time-efficient, and age-friendly. With high sensitivity and specificity, it is suitable for application in primary care, community, hospital, and home settings.

